# Detecting the Failures Nobody Sees: Applying Pareto Analysis and Failure Mode and Effects Analysis to Equipment Reliability in a Healthcare Simulation Institute

**DOI:** 10.7759/cureus.115428

**Published:** 2026-08-30

**Authors:** Pernel F Roberts, Melissa Remy, Ashlee Lavine, Rakesh Calton

**Affiliations:** 1 Clinical Foundations, Ross University School of Medicine, St. Michael, BRB

**Keywords:** failure mode and effects analysis, medical simulation, pareto analysis, quality improvement, risk priority number, simulation operations

## Abstract

Background

Hospitals have used failure mode and effects analysis (FMEA) for two decades to examine clinical processes before they fail, and healthcare simulation has served as a useful instrument for carrying out that analysis. The equipment inside the simulation center has attracted far less scrutiny. Audiovisual (AV) and manikin failures interrupt teaching, and when video capture is affected, they destroy the recording on which objective structured clinical examination (OSCE) grading and debriefing depend.

Methods

We analyzed a 24-month log of reported equipment incidents (N = 22) from the simulation institute of an offshore medical school using Pareto analysis, Fishbone (Ishikawa) diagrams with Five Whys questioning, and FMEA. The work formed the Define, Measure, and Analyze phases of a Define-Measure-Analyze-Improve-Control (DMAIC) quality improvement cycle. Severity anchors were set by institute leadership according to consequence; Occurrence was scored from observed frequency; and Detection reflected how each failure mode ordinarily comes to notice. The risk priority number (RPN) was calculated as the product of the three scores.

Results

AV and network problems accounted for seven incidents (31.8%) and manikin power or mechanical failures for six (27.3%), together making up 13 (59.1%) of the total. FMEA ranked capture failure during OSCEs at the top, with an RPN of 378, well ahead of manikin total shutdown at 140, even though the shutdown carried the maximum severity score. Detectability accounted for the whole of the difference. A shutdown stops the session and is obvious within seconds; a capture failure leaves the session looking entirely normal and comes to light only when someone asks for a recording that was never made.

Conclusions

Prioritizing equipment risk by severity alone would have ranked manikin shutdown as the highest risk, while leaving the more damaging failure unaddressed. Detectability deserves a place alongside severity in simulation operations improvement work. We set out the incident classification scheme, the scoring anchors, and a five-step workflow so that other programs can run the same analysis against their data.

## Introduction

On July 8, 2024, the video capture system in one of our simulation rooms failed during an objective structured clinical examination (OSCE). It recorded neither video nor audio. The encounter itself ran to completion without anyone noticing a problem, and only later did it emerge that the single record of that student's performance, the record used for debriefing and for grading review, did not exist. Over the following two years, the log accumulated several more incidents of the same kind.

Our institute is the dedicated simulation facility of an offshore medical university whose four-year United States-curriculum Doctor of Medicine (MD) program anchors all pre-clinical teaching in simulation. Students pass through five pre-clinical semesters of 15 weeks each, in cohorts of roughly 150 to 250, with three cohorts overlapping at any time, so the institute trains about 1,000 students a year. At that volume, working equipment is not a background convenience. It is a condition of delivering the curriculum at all.

Failures were, of course, familiar to our simulation operations (SimOps) staff, who fixed each one as it arose. What nobody had was a view across incidents. A capture failure during an OSCE and a manikin shutdown both interrupt teaching, but they are not the same kind of risk: the shutdown halts the room and cannot be missed, while the capture failure can sit undiscovered until someone goes looking for footage that is no longer there.

Quality improvement methodology in healthcare operations

Lean, Plan-Do-Study-Act, and Six Sigma dominate quality improvement practice in healthcare. Plan-Do-Study-Act suits small iterative cycles, while the Define-Measure-Analyze-Improve-Control (DMAIC) roadmap associated with Six Sigma suits problems that need systematic characterization before anyone intervenes [1]. Support services have been a productive area for improvement. A systematic review of Lean and Six Sigma in hospital pharmacy operations drew on 73 studies and reported reduced process variation, improved workflow, better inventory control, and fewer bottlenecks and failure modes [2]. Wider reviews of Lean Six Sigma in healthcare repeatedly identify DMAIC, failure mode and effects analysis (FMEA), visual management, and statistical process control among the tools associated with success [3], and there is evidence that these methods work well alongside simulation used as a modeling environment for testing countermeasures before implementation [4]. A simulation institute is, structurally, a support service of exactly this type, and its output is reliable equipment and an intact assessment record.

FMEA is well established in hospital settings

FMEA came into healthcare from engineering and is now a mainstream prospective risk tool, recommended by accreditation bodies as a way of finding how a process might fail before any patient is harmed [5]. Its reach is considerable. One systematic literature review gathered 158 healthcare FMEA papers published between 1998 and 2018 and sorted them into four groups: healthcare processes, hospital management, hospital informatization, and medical equipment and production [6]. A review confined to hospital wards examined 22 studies and found the work clustered in pediatrics and radiology, with nursing staff supplying most participants [7]. Medication safety has attracted a systematic review of its own [8].

The individual applications range widely. Teams have used healthcare FMEA to cut central line-associated bloodstream infections in a neonatal intensive care unit [9] and to improve hormone testing in the laboratory, where the highest-ranked failure turned out to be a delay after samples left the department rather than anything that happened during analysis [10]. Across all of it, the process under examination is some form of patient care. Institutions rarely apply the method to their own supporting infrastructure, and we could find no instance of it being applied to educational infrastructure.

The risk priority number (RPN) and its critics

The RPN, the product of Severity, Occurrence and Detection, is what FMEA produces and also what attracts most of the criticism. Shebl and colleagues asked two multidisciplinary hospital teams to conduct FMEAs of the same medication process and then tested the results against four validity criteria, including the trust's own incident database. Face validity held up. Content validity did not fare as well, since clinicians outside the teams identified failures both had overlooked, and on construct grounds, the authors questioned whether ordinal scores should be multiplied together at all [11]. Others have since proposed RPN formulations better suited to healthcare [12], and a substantial methodological literature now addresses the same weaknesses [13].

We accept these criticisms. They caution against reading an RPN as a precise quantity, which we avoid, but they support its use for setting priorities. Our own findings turn on the Detection term, which does work in a way that no severity-based ranking can reproduce.

Simulation and FMEA: two established directions, and a gap

Healthcare simulation has approached FMEA from two directions. Neither is the one taken here.

The first, and by far the more common, uses simulation as the instrument and a clinical process as the object. Davis and colleagues set out the methodology, running FMEA from in situ simulations to understand clinical risks and failures [14]; Nielsen and colleagues later showed that watching simulated scenarios directly surfaced more failure modes than brainstorming did [15]. Applications have followed steadily. Six in situ simulation-based clinical systems tests of intraoperative emergencies in robot-assisted thoracic surgery produced FMEAs identifying 28 latent safety threats, of which nine ranked as high priority [16], and quality improvement tools have been applied to prioritize and mitigate threats surfaced through in situ simulation of pediatric shock [17]. Around this topic sits a large literature on in situ simulation as a detector of latent safety threats in emergency departments [18], pediatric intensive care [19], operating theaters [20], and cardiac arrest response [21]. Throughout, the simulation center supplies the laboratory, while the clinical system supplies the specimen.

The second direction treats a simulator as a piece of engineering. Bartuska and colleagues combined FMEA with multi-criteria decision-making methods to rank failure modes in a central venous catheterization trainer and guide its redesign [22]. The object is one device at the design stage, not a working estate of equipment in daily use.

We are not aware of any published study that has applied FMEA to the operational equipment of a simulation institute itself: the manikins, capture platforms, networks, and workstations that carry the curriculum. Reversing the usual direction in this way is what the present study attempts.

Why the simulation institute deserves this attention

The argument for treating simulation infrastructure as a quality improvement target is educational before it is operational. Simulation-based medical education with deliberate practice outperforms traditional clinical teaching for skill acquisition [23], and much of that advantage rests on debriefing, which meta-analytic work identifies as a principal active ingredient [24]. Kolb's account of experiential learning explains the mechanism, describing skill as something that forms when concrete experience passes uninterrupted into reflective observation [25]. Equipment failure can break that sequence at any point. Capture failure disrupts this sequence at the worst possible time by erasing the record on which both reflection and assessment depend. If debriefing is where the educational benefit is realized, a silent capture failure has deleted the mechanism by which it was supposed to work, rather than merely inconveniencing the session.

Operations standards take a similar view, treating infrastructure, personnel, and process as inseparable parts of program quality rather than as conditions in the background [26], and accreditation standards for systems integration place operational governance and risk management among the marks of a strong simulation program [27].

We, therefore, set out to describe the equipment and audiovisual (AV) failure profile of a single institute across 24 months, to rank failure modes by risk rather than by frequency, and to produce both local interventions and a template that other programs could apply to their own data.

## Materials and methods

Study design and setting

The study was conducted at the Simulation Institute, Ross University School of Medicine, Two Mile Hill, St. Michael, Bridgetown, Barbados. This was a retrospective, single-center descriptive quality improvement study using routinely collected equipment incident data, forming the Define, Measure, and Analyze phases of a DMAIC cycle within an institutional quality and process improvement initiative. The institute holds accreditation from the Society for Simulation in Healthcare and works within an institutional commitment to total quality management and continuous quality improvement.

Equipment comprises five high-fidelity manikins; nine Harvey cardiopulmonary simulators (Laerdal Medical, Stavanger, Norway; originally developed at the Gordon Center, University of Miami, Miami, FL, USA); and 25 AV-equipped rooms, of which five are simulation rooms, five are debriefing rooms, and 15 are small-group learning rooms. All are connected to a vendor-supplied video capture platform used for live observation, debriefing, and grading review. The simulation rooms are laid out as emergency department bays, with a manikin on a gurney and a fixed routine in which students attach a cardiac monitor, an intravenous line, and a pulse oximeter. Small-group rooms are laid out as a physician's office, where a standardized patient meets eight students, and they were never built around that fixed routine. Manikins are maintained in-house by the simulation manager, whereas the capture platform is a separate vendor system covered by a scheduled-service contract.

Data source

The data come from a 24-month incident log kept by SimOps staff, covering June 2024 to May 2026 and containing 22 reported equipment and AV incidents. Records were extracted from the existing incident-reporting workbook, de-identified, and coded by the author team into nine failure modes. The source log held free-text descriptions rather than pre-set categories, so coding was done retrospectively. No student-identifiable data and no recordings were accessed.

Inclusion and exclusion criteria

All entries logged during the study period were eligible for inclusion. We included any reported failure of institute equipment or audiovisual infrastructure that occurred during, or in preparation for, a scheduled teaching or assessment session, irrespective of whether the session was disrupted and irrespective of how quickly the fault was resolved. We excluded entries recording scheduled preventive maintenance, planned software updates, or equipment relocation rather than a failure; entries relating to equipment outside the institute's inventory; entries describing facilities issues with no equipment or audiovisual component, such as room temperature or furniture; and duplicate entries describing the same event. Entries with insufficient description to identify the equipment involved were also excluded. No incident was excluded on the basis of severity, category, or reporter. All 22 entries logged during the period met these criteria and were analyzed.

Pareto analysis

Failure modes were ranked by frequency to establish which categories carried the bulk of reported incidents, based on the usual expectation that a minority of categories will account for most events.

Fishbone diagram and the Five Whys

The two commonest categories, AV and network problems and manikin power or mechanical failures, were taken forward for root cause analysis using a fishbone (Ishikawa) diagram with branches for Equipment, Process, People, and Communication. The Five whys questioning of the incident narratives traced AV and network failures back to instructor workstations approaching eight years of service, the lack of any standard pre-session AV self-test, and dependence on a separate vendor system for capture. Manikin failures traced back to cables routed through high-traffic areas during OSCEs, particularly in the classroom-style rooms, and to the absence of a cable inspection at prebrief. The author team carried out this analysis by reviewing incident narratives; it was not conducted as a live multidisciplinary workshop.

Failure mode and effects analysis

Each failure mode received scores for Severity, Occurrence, and Detection on a 10-point scale, and the RPN was calculated as their product. The scoring anchors are reproduced in full in the Discussion so that other programs can adopt or adapt them. The project protocol had originally set severity by event category, assigning one fixed score to each type of equipment failure. We refined this during the Analyze phase to a consequence-based scheme because the same technical fault carried very different consequences depending on when it occurred, and a category score could not tell those situations apart. Severity anchors were then set by institute leadership according to institutional risk tolerance, reserving the highest score for failures that postpone or disrupt a session and assigning lower scores in turn to non-capture of OSCE assessment data, non-capture during a training session, and all remaining outcomes. Occurrence followed observed frequency across the 24 months. Detection reflected how each failure mode ordinarily comes to notice, with session-stopping failures scoring lowest and silent capture failures highest. Detection scores were assigned by the author team and were not validated by a multi-rater process, a limitation consistent with documented concerns about Detection scoring even within full clinical teams [11].

Ethical considerations

This work forms part of an internal quality and process improvement initiative entitled Six Sigma Lean Project: Reducing Sentinel Events and Improving Equipment Reliability. The Ross University School of Medicine Institutional Review Board reviewed the initiative and approved it as exempt from Institutional Review Board review on December 9, 2025 (RUSMIRB exempt #2-IRB2025_02). No human participants were involved, and no experimental exposure was assigned to any individual. The units of analysis are equipment events and operational processes rather than people, and staff participated only in their ordinary operational roles. All incident records were de-identified before analysis. No student assessment data, examination performance records, or video recordings were accessed at any stage.

## Results

Pareto distribution

Table 1 sets out how the 22 reported incidents are distributed across categories. AV and network problems came first with seven incidents (31.8%), followed by manikin power or mechanical failures with six (27.3%). Between them, these two categories accounted for 13 (59.1%) of reported incidents, which is why we took both forward for root cause analysis. Logistics and delivery problems and documentation and process problems each contributed three (13.6%), instructor personal computer (PC) or laptop faults two (9.1%), and patient monitor faults one (4.5%).

**Table 1:** Pareto distribution of reported equipment and audiovisual incidents by category (N = 22, June 2024 to May 2026) Categories were derived from free-text incident descriptions, which were not pre-coded in the source log. AV: audiovisual; PC: personal computer

Category	Count	% of total	Cumulative %
AV/network	7	31.8	31.8
Manikin power/mechanical	6	27.3	59.1
Logistics/delivery	3	13.6	72.7
Documentation/process	3	13.6	86.4
Instructor PC/laptop	2	9.1	95.5
Patient monitor	1	4.5	100.0

Root cause analysis

Figure 1 shows the Pareto distribution graphically, with cumulative percentage plotted against category. Figure 2 shows the fishbone diagram for AV and network incidents, and Figure 3 the equivalent for manikin power and mechanical incidents. Neither fishbone analysis produced a dominant root cause in every branch: the People branch yielded nothing decisive for AV and network incidents, nor did the Communication branch for manikin incidents.

**Figure 1:**
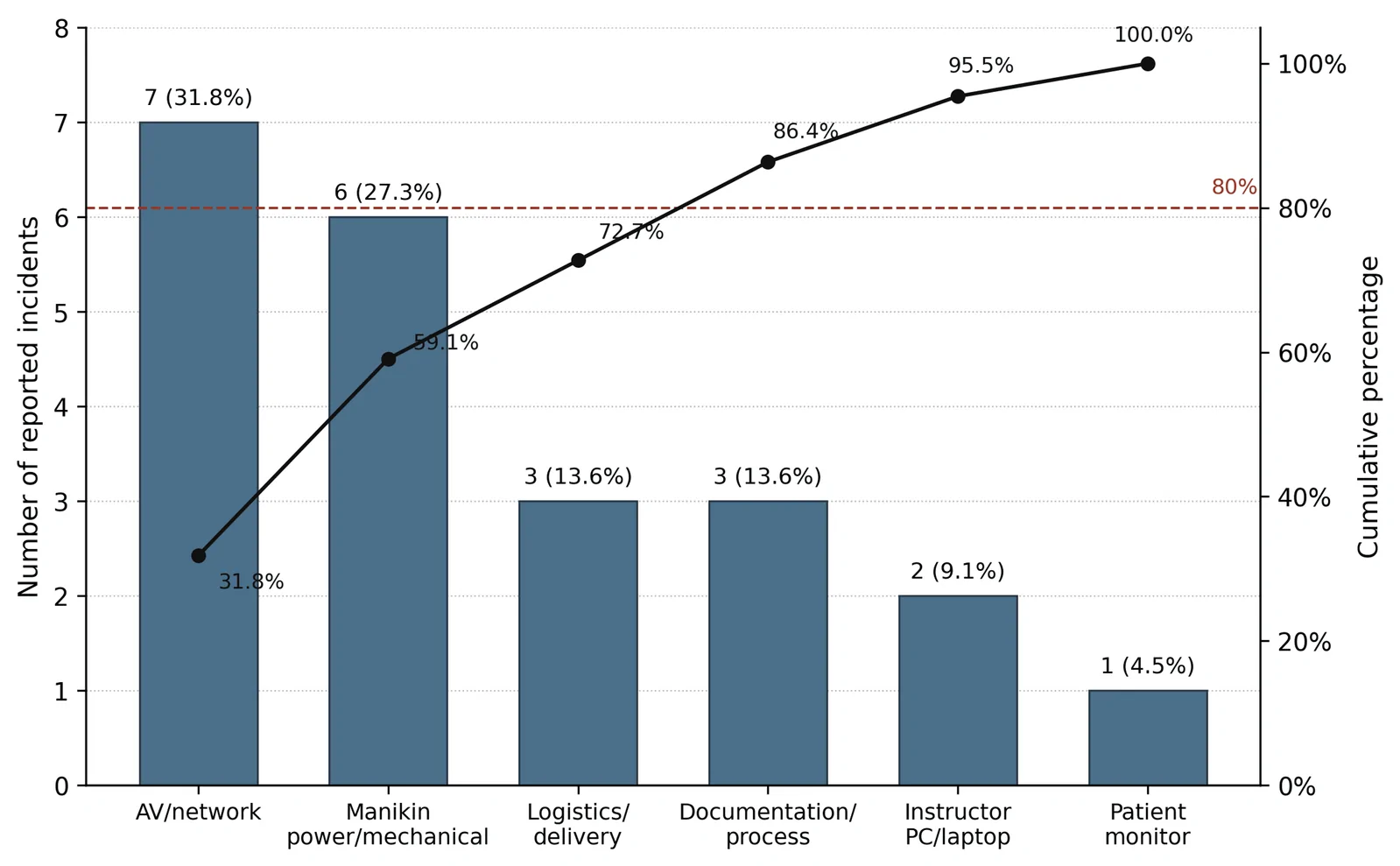
Pareto distribution of reported equipment and audiovisual incidents Bars show incident counts by category with percentage of total; the line shows cumulative percentage. The dashed line marks the 80% threshold. AV: audiovisual; PC: personal computer

**Figure 2:**
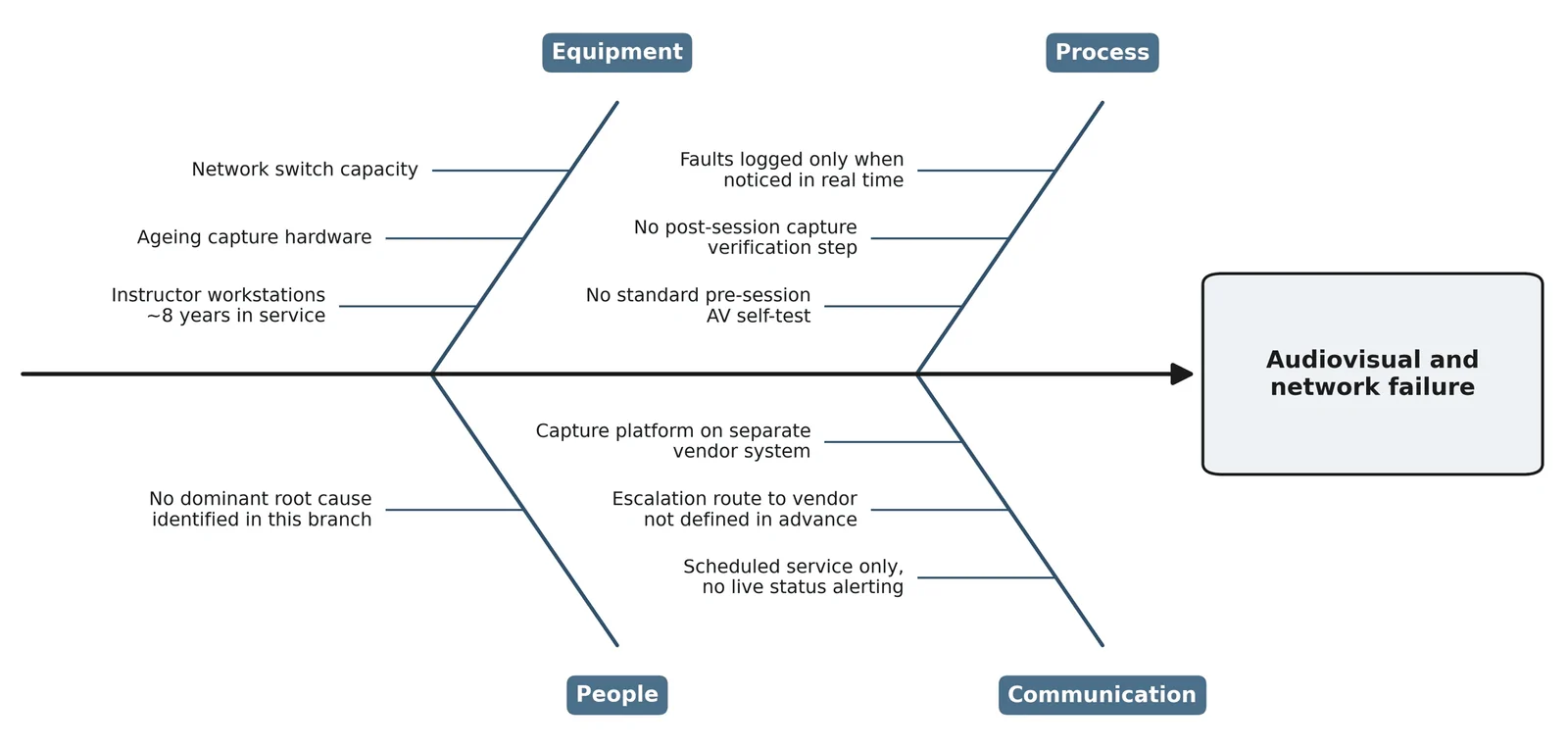
Fishbone (Ishikawa) diagram of contributory factors in audiovisual (AV) and network failures

**Figure 3:**
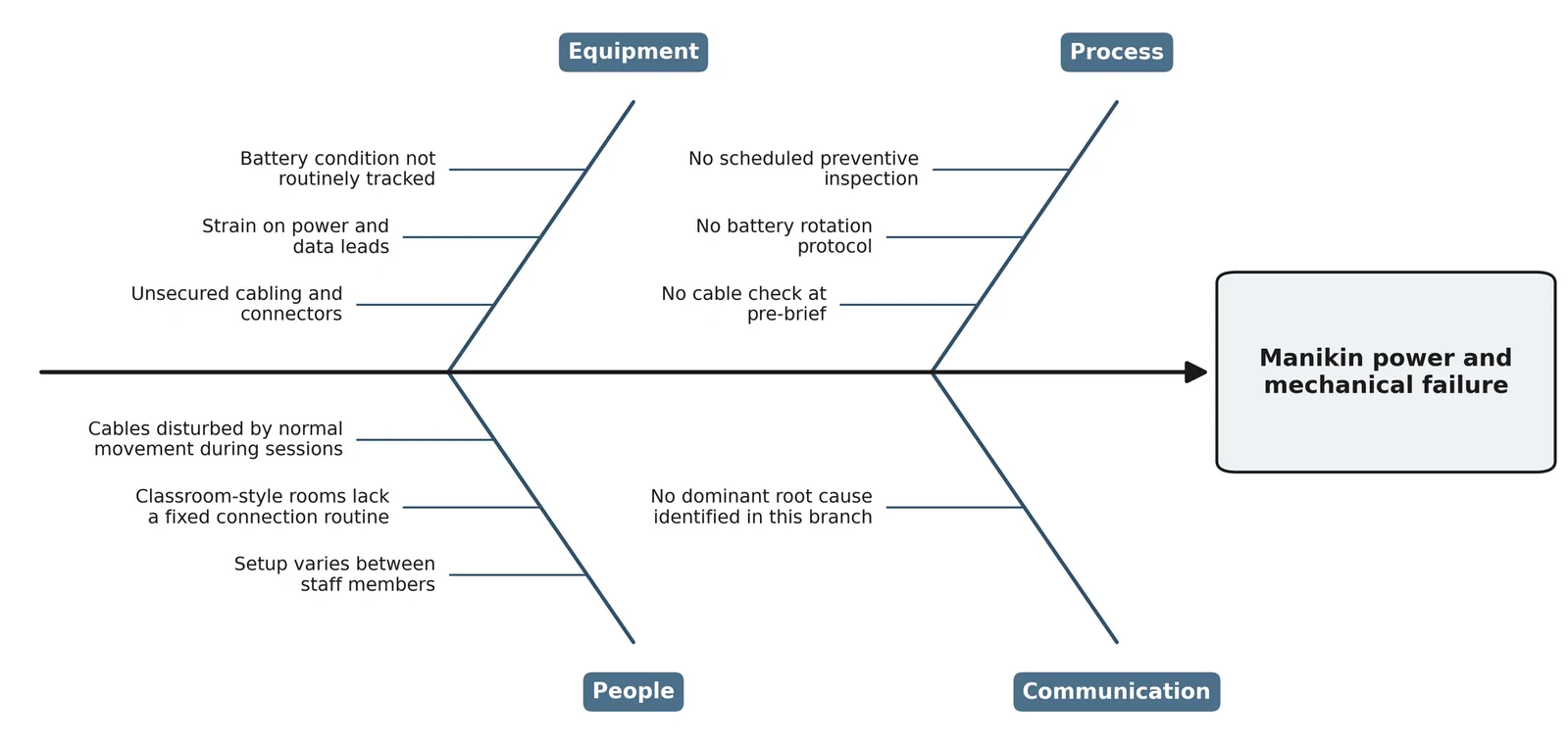
Fishbone (Ishikawa) diagram of contributory factors in manikin power and mechanical failures

Failure mode and effects analysis

Table 2 presents the FMEA results ranked by RPN. Capture failure during OSCEs came first at 378. Manikin total shutdown, despite carrying the maximum severity score, came fifth at 140. Detection rather than severity or occurrence explains the gap. A shutdown halts the session outright and is recognized within moments, which earns it the lowest detection score available. A capture failure leaves the session untouched: students carry on, facilitators observe as planned, and nothing at all signals that the recording has failed. The problem surfaces days later, when someone requests footage for grading or debriefing.

**Table 2:** Failure mode and effects analysis summary for nine failure modes, ranked by RPN RPN: risk priority number; Sev: severity; Occ: occurrence; Det: detection; OSCE: objective structured clinical examination; AV: audiovisual; PC: personal computer RPN = Severity × Occurrence × Detection, each scored on a 10-point scale.

Failure mode	n	Sev	Occ	Det	RPN	Recommended action
Capture failure, OSCE	3	9	6	7	378	Mandatory post-session capture-verification checklist
Documentation/process error	3	6	6	5	180	Digitize chart workflow; turnover checklist
General AV signal failure	3	9	6	3	162	Daily AV self-test; vendor escalation contact
Capture failure, training	1	8	3	6	144	Include capture check in setup checklist
Manikin total shutdown	4	10	7	2	140	Cable management and strain relief; prebrief cable check
Manikin partial malfunction	2	6	5	4	120	Battery rotation protocol; scheduled inspection
Logistics/delivery delay	3	6	6	2	72	Visual cue protocol via camera; delivery SOP
Instructor PC/laptop freeze	2	6	5	2	60	Scheduled PC re-imaging; backup laptop on standby
Patient monitor fault	1	6	3	3	54	Secure Ethernet cabling; facilitator training

Documentation and process errors ranked second at 180, sharing the same poor detectability, since a charting error may go unnoticed until a discrepancy forces attention. General AV signal failures such as surround-sound dropouts ranked third at 162; staff usually notice these in real time, but a high severity score lifted them above more frequent modes. Capture failure during training sessions ranked fourth at 144, scoring below its OSCE equivalent on both severity and occurrence, although the technical fault is identical. The remaining modes fell into the lower half of the ranking through some combination of lower severity, lower frequency, and faster detection.

## Discussion

The highest-priority equipment risk at our institute turned out to be a problem of Detection rather than of Severity, and the way our rooms are built helps explain why. In the emergency department bays, everyone in the room actively works through the routine of attaching a monitor, an intravenous line, and a pulse oximeter, so when a disconnection occurs, it interrupts a visible sequence and prompts a reaction. The small-group rooms were never designed around that routine. When a student disconnected a manikin during an examination in one of them, the cause was ordinary movement in a space unsuitable for the equipment, not a fault in the equipment. In both room types, though, the disconnection announces itself the moment it happens.

Video capture behaves differently. Our maintenance contract covers periodic service, training, and troubleshooting, and the simulation manager repairs manikins directly, which probably accounts for how quickly manikin problems are resolved. Capture operates on a different vendor system, and scheduled service contracts do not address the core issue: capture failures go unnoticed unless someone is watching the rooms. Vendor responsiveness is useless for unnoticed problems. That is why the highest RPN in our analysis attaches not to the most severe failure but to the one that stays invisible until a recording is requested and found never to have been made.

The significance of the RPN

Our results give a concrete demonstration of what the Detection term contributes and of why the RPN remains useful in practice despite its mathematical weaknesses [11-13].

Two failure modes competed for the top position. Manikin total shutdown occurred four times, more often than anything else, and carried the maximum severity score because it stops a session outright. Capture failure during OSCEs occurred three times and scored one point lower on severity. Rank by frequency and shutdown wins. Rank by severity and shutdown wins. Ranked by severity multiplied by occurrence, a common shortcut, and shutdown still wins, 70 to 54. The order reverses only when detectability enters, because shutdown is the most detectable mode in the set and silent capture failure the least. The RPNs come out at 140 and 378.

That reversal is not an artifact of the arithmetic. It describes something real about how organizations respond. A failure that announces itself has already triggered the response mechanisms that exist to address it, so it is half solved before anyone decides to act. A quiet failure consumes no attention until the damage becomes permanent. In assessment, the difference is decisive: a manikin shutdown during an OSCE produces a disrupted encounter that can be rescheduled, whereas an undetected capture failure produces an encounter that appears to go perfectly and is found to be unrecoverable only when grading starts.

For other simulation programs, the implication is uncomfortable. Any prioritization exercise that stops at frequency or severity will systematically under-rank exactly those failures whose consequences are hardest to undo. In our setting, Detection was not a refinement of the analysis. It was the analysis.

A replicable template for other simulation programs

Our failure modes reflect one institution's vendors, hardware, software, staffing, and workflows, and a center with different infrastructure should expect a different profile. What travels is the method rather than the ranking. Table 3 provides our anchors in full, and the five steps below describe the workflow in a form other programs can follow.

**Table 3:** Scoring anchors used in this study, offered as a starting template for adaptation Occurrence anchors are frequency bands specific to a 24-month window and a program of this size. Other centers should rescale them to their observation period and activity volume. OSCE: objective structured clinical examination

Dimension	Score	Anchor definition
Severity	10	Failure causes session postponement or disruption
Severity	9	Failure causes non-capture of OSCE assessment data
Severity	8	Failure causes non-capture during a training session
Severity	6	All other failure modes
Occurrence	7	Four occurrences over 24 months
Occurrence	6	Three occurrences over 24 months
Occurrence	5	Two occurrences over 24 months
Occurrence	3	One occurrence over 24 months
Detection	7	Silent; discovered only when the output is later requested
Detection	6	Silent during the session; discovered at next system check
Detection	5	Discovered when a downstream discrepancy arises
Detection	4	Partial or intermittent symptom during the session
Detection	3	Noticed in real time by staff monitoring the session
Detection	2	Stops the session; recognized immediately by everyone present

Log Rather Than Resolve

Record every technical failure in one structured log, including the ones fixed in two minutes. The minimum fields are the date and time of occurrence, room, equipment involved, a free-text description, time of detection, who detected it, time to resolution, session type, and whether the session was affected. Time of detection is the field most often left out, and it is the one on which everything else in this method rests.

Code Retrospectively Into Failure Modes

Staff will record free text more reliably than they will pick a category, so let them write and code afterwards. Use at least two coders. Split modes that carry different consequences even when the underlying fault is identical, since capture failure in an OSCE and in a training session is the same defect at different stakes, and keeping them apart is what allows severity to discriminate.

Determine the Volume Before Setting Priorities

Use Pareto analysis to see where incidents concentrate, then use that to choose which modes deserve root cause analysis. Do not allow it to set priorities by itself.

Score Against Pre-agreed Anchors

Agree on severity anchors with program leadership before any scoring begins so that severity reflects institutional risk tolerance rather than whatever the scorer thought that morning. Anchor severity to consequence rather than to equipment category, since one fault can matter very differently in different sessions. Score Detection with several independent raters and report their agreement.

Act on Detectability First

When a high RPN comes from a high Detection score, the first intervention should create a point of detection rather than try to prevent the failure. Putting a verification step where a silent failure would otherwise slip past turns an invisible risk into a visible one, and that is a far smaller and cheaper change than engineering out the fault.

Planned improvement cycle

Two interventions follow from the Pareto and FMEA findings and will form the Improve phase. The first is a mandatory post-session capture check, in which a SimOps team member confirms that a recording exists before releasing the room, starting with OSCEs because the assessment stakes are highest there. The second is a cable-securing protocol for manikin connections in the classroom-style rooms, covering floor securing and a check at prebrief, which addresses the root cause identified through Five Whys. Both will run as a two-semester pilot alongside continued incident logging, which will be coded consistently with this baseline, with monthly review of incident counts and checklist adherence. We will report the Study and Act stages once that measurement period has finished.

Limitations

This is a single-institution study built on 24 months of data and 22 reported incidents, and its findings should be read as a local prioritization baseline rather than as a failure rate anyone else could apply. With numbers this small, we cannot identify trends and recurring patterns with confidence.

Everything here rests on a voluntary technology issue log, so underreporting, inconsistent categorization, and variable reporting habits may all have shaped the priorities we identified. Given how much activity the institute supports, it is likely that the log undercounts what actually happened, and readers should treat these figures as reported incidents rather than as all incidents. That limitation is itself part of the case for the first step of the template above.

Occurrence scores came from the same incident counts that produced the Pareto distribution, so the two analyses are not statistically independent. Severity followed criteria set in advance by the institution, but Detection scores rest on the author team's judgment and were not checked against independent raters, which echoes documented concerns about Detection scoring in FMEA generally [11,12]. Fishbone and Five Whys themes likewise came from reviewing written narratives rather than from a workshop with frontline staff, whose direct experience might well have surfaced causes that the written record does not contain.

Finally, this report covers only Define, Measure, and Analyze. No post-intervention period has been completed, and the effect of the planned interventions remains unknown.

## Conclusions

Hospitals have used failure mode and effects analysis for years, and healthcare simulation has applied it in two directions: outward, to study clinical processes, and inward, to the design of individual training devices. Turning it instead on a simulation institute's own working equipment produced an unexpected result. The highest-priority risk was the least detectable failure, a capture failure during an OSCE, rather than the most severe failure; this failure can go unnoticed until the recording it should have produced is needed and found to be missing. Ranking by severity or by frequency would have reversed that priority and left the risk in place.

Detectability therefore deserves explicit weight in simulation operations improvement work, and the first move against a silent failure should be to create a point of detection rather than to engineer out the fault. We offer the anchors and a five-step workflow as a starting point for other simulation programs, and we will report what the resulting interventions achieved once a full measurement period has passed.
